# Perceived challenges in implementing halal standards by halal certifying bodies in the United States

**DOI:** 10.1371/journal.pone.0290774

**Published:** 2023-08-31

**Authors:** Omar A. Al-Mahmood, Angela M. Fraser

**Affiliations:** 1 Department of Veterinary Public Health, College of Veterinary Medicine, University of Mosul, Mosul, Iraq; 2 Department of Food, Nutrition, and Packaging Sciences, Clemson University, Clemson, SC, United States of America; University of Ilorin, NIGERIA

## Abstract

Islamic dietary laws inform halal standards, defining which foods are halal (lawful and permissible). Many halal foods are produced in non-Muslim majority countries increasing the likelihood they might be haram (prohibited). Halal certification is one way to operationalize halal standards, hence protecting Muslim consumers. At present there is no unified halal standard guiding halal certification. The aim of this study was to determine the perceived challenges in implementing halal standards in the United States. Semi-structured in-depth interviews were done with a representative from 6 of the 11 halal certifying bodies (HCBs) in the United States. All reported their role was to verify food safety records for compliance with government regulations but not to monitor food safety practices. Two main issues—forged halal certificates and expired halal logos were identified as significant issues. Three HCBs stated there is no problem with having multiple halal standards, but all believed it is necessary to have one universal halal standard with minimum standards followed by everyone. The findings of this study can be beneficial to the U.S. halal industry as it highlights the challenges and issues they face.

## Introduction

The demand for halal food is increasing worldwide [1, 2]. Halal, an Arabic word, means an act, object, or conduct permissible or lawful, whereas haram means prohibited or unlawful. Both words apply to food consumed by Muslims. All halal foods must be produced in compliance with Islamic dietary laws.

Many halal foods and ingredients are produced in non-Muslim majority countries [3] possibly increasing the likelihood they might be contaminated with haram ingredients, such as common ingredients derived from pigs (e.g., gelatin, enzymes, glycerin, lecithin, L-cysteine, and mono- and diacylglycerols) because of a lack of awareness of their haram status. If a haram ingredient is present even in a trace amount, the finished product is haram so cannot be consumed by a Muslim consumer. Consequently, halal products manufactured in non-Muslim countries require more oversight to maintain their halal status compared to products manufactured in Muslim countries [4].

Laboratory analytical methods are often used to detect the presence of haram food ingredients within the limits of their detection. However, the halal status of meat and poultry products cannot be analytically measured as halal standards for animal foods can only be determined through visual inspection [3]. For these and other reasons, it is necessary for food manufacturers, producers, and exporters to receive halal certification from a reliable halal certifying body (HCB) [4]. Halal certification is the process of certifying products (food and non-food) for compliance with Islamic dietary law [5]. Certification is typically used to enhance the marketability of halal foods, particularly to Muslim consumers. Halal certification also helps Muslim consumers know they are consuming a true halal product [6, 7].

Prior to the 20^th^ century, Muslims prepared and consumed most of their food without relying on imports. Since the 1970’s, food has been increasingly imported from non-Muslim majority countries into Muslim-majority countries which therefore need halal certification [8]. In addition, as the Muslim population increases in non-Muslim majority countries, the criticality of halal certification will become more pronounced.

In the early 1980’s, the first HCB was established to monitor and enforce halal standards in food [9]. Globally, there are now more than 400 HCBs [10], with 11 operating in the United States. Halal certification is sometimes issued by a national government, such as in Malaysia, whereas in other countries the certification is endorsed by one or more private Islamic organizations. HCBs follow a halal standard recognized as in accordance with Islamic dietary laws. However, different halal standards, due to differences in the interpretation of Islamic dietary laws, are used by various Islamic agencies and certified within an individual HCB as a principle of judgement in addressing daily halal processes. Halal standards have been developed that have been accepted legally by more than one country (e.g., The Standards and Metrology Institute for the Islamic Countries (SMIIC) and GCC Standardization Organization (GSO)) or by governmental agencies (e.g., Department of Islamic Development Malaysia (JAKIM), Islamic Religious Council of Singapore (Muis), Majelis Ulama Indonesia (MUI), and the Central Islamic Committee of Thailand (CICT)).

Halal certification typically includes two stages: 1) online application and pre-screening products and its raw materials/ingredients and 2) an on-site audit verifying all documents regarding halal raw materials as well as inspecting the entire production process by inspecting the production facility, particularly if both halal and haram products are run in the same facility, to ensure full compliance with halal standards [11]. Post-audit activity is conducted by an approval panel (members of the certification organization and outside religious leaders) that decide if a halal certificate will be issued. Food manufacturers and producers are also required to have in place additional food safety systems, such as Hazard Analysis Critical Control Point (HACCP), Good Manufacturing Practices (GMP), and Sanitation Standard Operating Procedures (SSOPs) [12] as Islamic dietary laws also require halal foods to be safe, clean, and hygienic (Tayyab) [13].

The lack of a unified halal standard due to different Islamic schools of thought has led to different interpretations and application of Islamic dietary law [14]. For example, three Islamic schools of thought (i.e., Imam Abu Hanifah, Imam Malik, Imam Ahmed) require the invocation of the Tasmiya at the time of slaughter whereas this is not required by Imam Al-Shafi another school of thought. Halal standards also vary among countries and sometimes within a country [15]. Issues associated with the lack of one universal halal standard include no global benchmark, higher production costs because of a mix of national halal standards, and risk of using a permissible method of slaughtering by one halal standard that may not be accepted by other halal standards (e.g., mechanical slaughter and animal stunning) [16]. Although several attempts have been made to harmonize halal standards among Islamic states, to date none of these attempts have been successful [15].

The aim of the study was to determine the perceived challenges in implementing halal standards obtaining preliminary answers to the following four research questions from the perspective of the HCBs.

1. What are the similarities and differences in the certification process among the HCBs in the United States?
2. How do HCBs ensure implementation of food safety practices in the halal food industry?
3. What are the perceived issues associated with applying different halal standards to the halal industry in the United States?
4. What would be the challenges of unifying and applying one halal standard to the halal food industry in the United States?

## Methods

The Institutional Review Board at Clemson University approved the research protocol of this study on June 7, 2019. Data collection began after approval was received.

### Target population and sample design

No specific database (list) was available that identified all HCBs in the United States. To construct a list of HCBs, an online search string was done through a combination of keywords and Boolean operators that has indexed a large part of the world’s websites (library database) to maximize the number of HCBs and to ensure that all addresses and contact information of the U.S. HCBs was obtained. The online search yielded 11 HCBs in the United States, with all issuing halal food certification. To confirm that every HCB included in this study had a U.S. trademark registration, phone calls were conducted. These 11 HCBs served as the sampling frame for this study.

### Recruiting participants (HCBs)

A semi-structured in-depth interview was conducted by telephone with participating HCBs (i.e., with the firm’s managing director) [17]. The 29 interview questions were structured to allow us to answer the four research questions. A letter of recruiting script (email or hard copy by mail) was sent to all 11 HCBs describing the aim and objectives of the study and conditions for participation in May 2019. Only participants who completed the confirmation letter could participate in the study. Subsequently, the informed consent transcript was sent to the HCBs that agreed to participate. Before data collection began, the HCBs director provided written consent through the mail. A copy of the interview questions was sent to each HCB before the actual interview date to allow the interviewee time to prepare their responses. All participants were informed the interview would take between 40 and 60 minutes. One day before the telephone interview, a reminder email was sent to each participating HCB.

### Interview and data collection

Two individuals conducted each interview, one led the interviews, and one took notes. The interviews were conducted in English between June and July 2019. The interviewer used a moderator’s guide created by the research team to ensure consistency across interviews. Interviewees were reluctant to give permission for audio-recording thus the interviews were hand recorded.

### Data coding and analysis

The interview notes were transferred to a Microsoft Excel worksheet (Microsoft 365, WA, USA), creating a single column consisting of all responses for each interview [18]. The initial step of analysis was assigning responses into meaningful units (based on the 29 interview questions). Some questions yielded 2–3 themes. The next step was to formulate themes for each of the four parts of the interview [19, 20]. A constant comparison approach was used to identify themes [20].

## Results

### HCB characteristics

**Fig 1:**
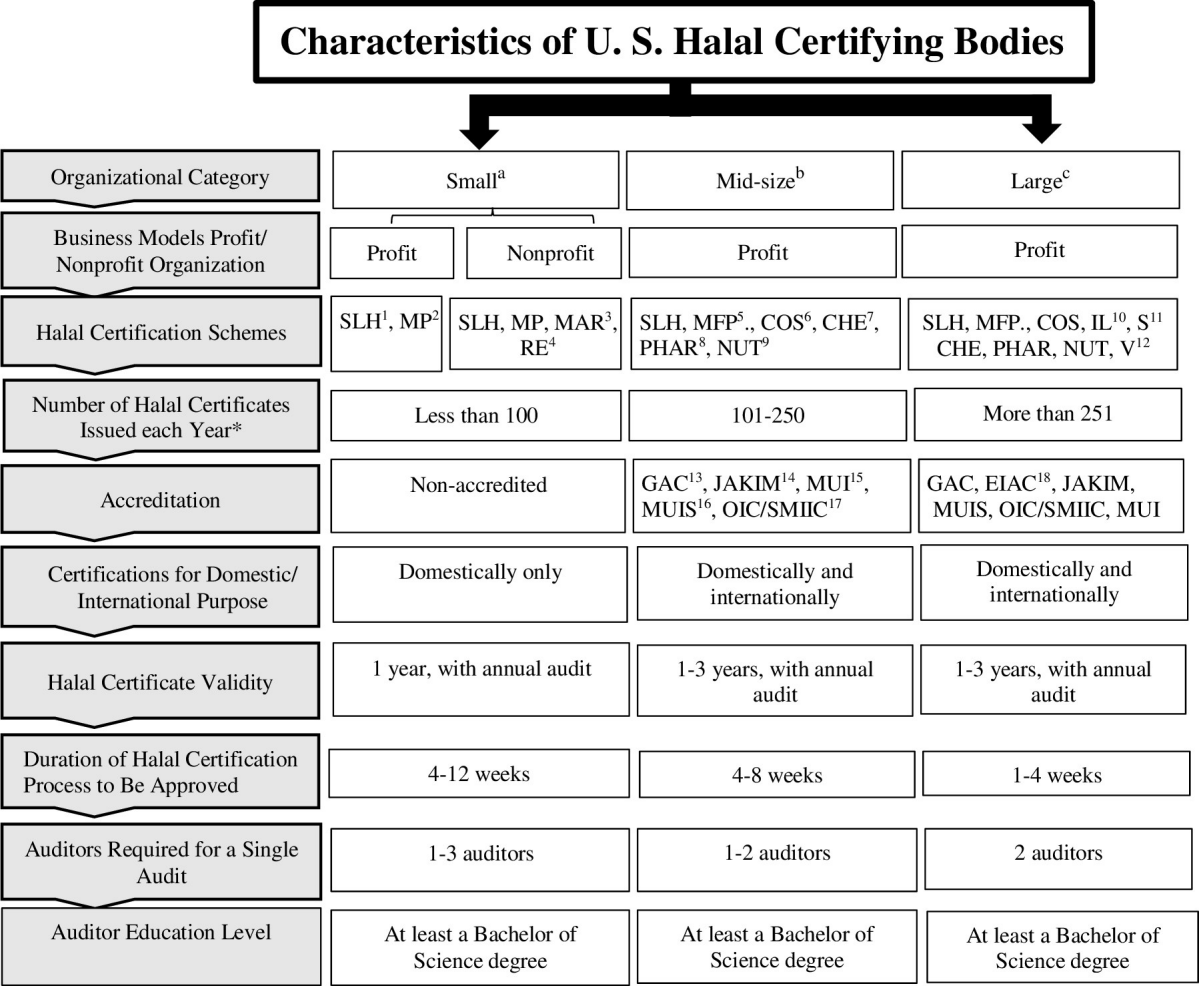
Classification scheme used to categorize HCBs in the United States. ^a^Small HCB could be profit or non-profit businesses, non-accredited, certified meat plants, markets, and restaurants (domestically only); it issued less than 100 halal certificates per year. ^b^Mid-size HCBs were profit businesses, accredited by overseas bodies, certified six schemes for the domestic and international purpose; it issued between 101–250 halal certificates per year. ^c^Large HCBs were profit businesses, accredited by overseas bodies, certified nine schemes for domestic and international purpose; it certified more than 251 halal certificates per year. *: Some halal certificates include hundreds of certified items in one document (one certificate for each company), ^**1**^SLH: Slaughterhouse, ^**2**^MP: Meat processing plant, ^**3**^MAR: Markets, ^**4**^RE: Restaurants, ^**5**^MFP: Meat and food processing plant, ^**6**^COS: Cosmetics, ^**7**^CHE: Chemicals, ^**8**^PHAR: Pharmaceuticals, ^**9**^NUT: Nutraceuticals, ^**10**^IL: Industrial lubricants, ^**11**^S: Sanitizers, ^**12**^V: Vaccines, ^**13**^GAC: GCC Accreditation Center, ^**14**^JAKIM: Department of Islamic Development Malaysia, ^**15**^MUI: The Council of Indonesian Scholars, ^**16**^MUIS: Islamic Religious Council of Singapore, ^**17**^ATC/SMIIC: Accreditation Technical Committees/The Standards and Metrology Institute for the Islamic Countries, ^**18**^EIAC: Emirates International Accreditation Center.

All 11 HCBs in the United States were classified into one of three categories (small, mid-size, and large). The classification system was based on (1) their business model (profit/non-profit); (2) halal certification scheme; (3) number of halal certificates issued each year; (4) accreditation (accredited/non-accredited by overseas bodies); and (5) certification for domestic/international purposes. After contacting all HCBs (N = 11) in the U.S., six agreed to participate in this study (small = 3, mid-size = 2, and large = 1) (Fig 1). Regarding the non-participating HCBs, the online search and phone call showed that all five were categorized as mid-size.

### Similarities and differences among the U.S. HCBs

All HCBs agreed the source of halal standards is the Holy Quran and Hadith (i.e., the traditions of Prophet Mohammad (PBUH), Messenger of Allah). All used a similar approach to certification, conducting on-site audits (annual audits) to verify all documents regarding halal raw materials as well as inspecting the entire production process to ensure compliance with halal standards. All confirmed that the time required for the process of issuing the halal certificate, from receiving the application to approving the halal certificate, ranged from 1–2 months. Lastly, all confirmed they had at least one auditor, all auditors were required to have a Bachelor of Science degree. The main difference between small HCBs and mid-size and large HCB was the use of different halal standards. Small HCBs applied their own halal standards (most stringent standard) compared to mid-size and large HCB (e.g., GSO, Malaysian standard, and SMIIC). For example, stunning before slaughter and the slaughterman being from the People of the Book (Jews and Christians) was accepted as a standard by mid-size and large HCBs that participated in the study but not by small HCBs.

### Halal certification and food safety practices

Nearly all (5/6) stated the federal government (USDA/FSIS or FDA) should monitor implementation of food safety systems (HACCP plan, SOP, GMP) in halal food manufacturing plants not the HCB. One small HCB had a different opinion, stating the HCB should be responsible for making sure halal food products are safe and free from any harmful substances (i.e., physical, chemical, and biological hazards) to be considered halal. All reported the HCB’s role is to verify all records and documents regarding food safety practices are in place and in compliance with government regulations as any food safety violations recorded by the HCB auditor could lead to non-conformity with halal standards. One small HCB stated they also verify food safety practices in halal food retailers (butcher shops) and restaurants according to each state’s public health department requirements.

### Issues and challenges of applying different halal standards

Opinions varied about the issues and challenges of applying different standards. The large and mid-size HCBs (3/6) reported that using different halal standards (e.g., GSO, Malaysian standard, and SMIIC) is important to meet the halal standards of the importing countries. In the U.S. domestic markets, the large and mid-size HCBs applied their own standards, consistent with the SMIIC standards. The three small HCBs implemented their own standards (e.g., Halal Product Integrity Protection, Halal Monitoring System—those responsible for ensuring that the halal system is functional and monitored) domestically (U.S. markets) in slaughterhouses and meat processing plants. These halal standards were created by each of these small HCBs using their own interpretation of the Quran and Hadith. All also stated that forged halal certificates and expired halal logos were a problem in the U.S.

### Challenges to unifying and applying one comprehensive halal standard

Four HCBs reported that applying multiple halal standards is not the reason for the use of various halal logos in the U.S. Each certifier has its own logo and respective brand, which is perceived to be important to be competitive. Two (small HCBs) had different opinions, stating many countries outside of the United States (mostly Middle Eastern and Asian countries) have their own halal standards that inform what halal logo is to be used. Nearly all (5/6) reported it is impossible at present to have one halal logo used by all U.S. HCBs. Only one certifier (small HCB) had a different opinion. He stated it could work if every certifier diligently met the same halal standard. Three reported that the lack of one unified halal standard is increasing production costs and complexity for the HCB and exporting companies. All believed that some halal standards are more flexible (less stringent) than others. The three non-accredited HCBs (small-sized) were not following any overseas halal standards.

All believed it was necessary to have one universal minimum set of halal standards followed by everyone. Five reported the possibility of establishing a competent organization devoted to maintaining and policing the halal standard in the United States. One stated there is no possibility to apply one comprehensive halal standard. Four believed that the OIC or International Halal Integrity (IHI) Alliance could develop one global unified halal standard. In addition, nearly all (5) stated that the big challenge was to establish a national halal hub in the U.S.

## Discussion

Although halal certification in the U.S. is not mandatory for manufacturers to produce halal products, most observant Muslim customers want to be assured food is compliant with halal standards [21, 22]. HCBs serve an important role in verifying the halal status of food products. The aim of this study was to answer four research questions to help us determine the perceived challenges of implementing halal standards in the U.S. market.

The first question aimed to answer: What are the similarities and differences among the U.S. HCBs? Interview findings showed all halal standards were informed by the Holy Quran and Hadith especially in term of the essential legislations, such as all halal foods and beverages must be free from pork and its derivatives and alcohol. The main point of difference was the interpretation and application of these halal standards. Halal standards may differ slightly as there are different Islamic schools of thought. For example, the most complete sticking of the animals is cutting the four parts which are the throat, esophagus, and two jugular veins (www.islamweb.net, fatwa: 62087). However, the four traditional Sunni Muslim jurists (Imam Abu Hanifah, Imam Malik, Imam Ahmed, and Imam Al-Shafi) representing different Islamic schools of thought vary regarding the condition of the animal sticking. Imam Abu Hanifah says slaughtering is acceptable by cutting three parts without specification, whereas Imam Malik says cutting off the throat and two jugular veins is acceptable. Imam Ahmed, and Imam Al-Shafi further differ by saying that the slaughtering is sufficient by cutting the throat and the esophagus only (www.islamweb.net, fatwa: 13939).

Another difference is some U.S. HCBs accredited by accreditation agencies (e.g., Gulf Accreditation Center) shared that accreditation makes one competent to certify halal for products or services [23]. However, not all HCBs, particularly the small ones, seek accreditation because it is not required for local halal production and distribution in the U.S., and it is expensive. Three HCBs (mid- and large-sized) reported that accreditation is essential as most accreditation bodies are recognized by Muslim countries importing halal products. Some Muslim countries (e.g., Malaysia, Indonesia, Pakistan, and Arab Gulf Countries) have a filtering process at the entry points for food products, especially meat and meat products entering the country to ensure consumable goods are halal certified [24]. However, small HCBs mainly certify slaughter and meat processing plants for domestic use because meat has the most halal restrictions that need to be verified by Islamic law.

The last difference is the mid- and large-sized HCBs offered more certification schemes compared to small HCBs as they included other food (not just meat) and non-food products (chemical and cosmetics) to meet the needs of Muslim consumers while small HCBs limited their scope to certifying only food products especially the meat and meat products. The one non-profit HCB in the sample also certified halal markets and restaurants to increase confidence of consumers across the food chain and achieving the concept of halal from farm-to-table guided by Islamic dietary law. Syed Marzuki (2012; 2016) reported the importance of Muslim consumers knowing how food is processed and prepared, even in a restaurant setting, to boost confidence in its halal status and to have ‘peace of mind’ [25, 26].

The second question was: What is the relationship between U.S. HCBs and food safety practices in halal food industries? Similar opinions were reported that the USDA and FDA have excellent programs to ensure implementation of food safety practices. HCBs duplicating this activity would cost the halal industry and consumers more without generating any additional benefit. During the on-site audit, if the auditor(s) noted a food safety issue (violation), considered to be non-compliant with halal standards, operators were required to correct it before issuing the halal certificate. For example, one HCB recorded a violation of food safety that occurred in a meat processing plant when raw meat was stored with finished products (ready-to-eat food) on the same shelf in the refrigerator. Multiple studies have emphasized the importance of evaluating food safety practices during halal food production and certification suggesting that food safety must be a condition for a food to become halal [13, 27–31]. Moreover, because of the well-developed food safety regulations in the U.S., halal foods are typically believed to be hygienic and safe. For example, one HCB interviewee indicated that one of their clients stated halal foods produced in the U.S. are more popular worldwide due to its quality and safety.

The third question was: What are the issues and challenges of applying different halal standards to the U.S. halal industry? Based on interview responses, the halal food industry, like most industries, seeks efficiency in doing its work. However, most HCBs focus on exports, which are based on each importer’s halal standards. Because importing countries vary in their required standards, they have to use multiple halal standards to meet the requirements of different countries. This becomes a challenge for the halal meat industry as Muslim consumers have different acceptance of halal standards. As related to meat slaughter, examples include that the name of God (Allah) should be recited before every single animal slaughtering or not; should the slaughterman be a member of People of the Book or a Muslim slaughterman; should the animal be stunned or not; and should there be a mechanical slaughter or hand slaughter. The halal food industry works with HCBs to allow them to apply the most appropriate standards. For example, some HCBs follow a specific halal standard that allows the slaughterman to be from the People of the Book (e.g., a Jewish religious slaughterman) to make an agreement with a kosher certifier to sell some parts of the carcass as kosher and others as halal. This makes it challenging for Muslim consumers to determine the standards with which the product was certified.

Small HCBs create their own halal standards (most stringent standard) according to their Islamic school of thought. This is done because some Muslim consumers do not accept some halal standards due to its conflict with their school of thought. Furthermore, similar to other countries, forged halal certificates and the use of expired halal logos also occur in the U.S., which not only decreases consumer confidence [32, 33] but increases the burden of all stakeholders to verify certification. Hence, one HCB stated that the responsibility of ensuring and tracing halal certificate rests with the certifier, so any expired or non-renewed certificate must be disclosed to the public to increase awareness in the Muslim community.

The fourth question was: What are the challenges to unifying and applying one halal standard in the United States? The halal logo is one of the most important factors in place to prove a meat is halal [34]. Most HCBs agreed that lack of a universal halal standard is not the reason for having different halal logos in the U.S. The primary reason is competition between HCBs. Each HCB has its own logo and brand to achieve commercial success. In contrast, two small HCBs shared concerns about having different halal logos because Muslim consumers need to further research the logo to prevent eating doubtful foods. In addition, several studies reported that multiple halal logos confuse consumers as they lacked information about the actual halal logo [35–37]. Nearly all HCBs agreed it was nearly impossible to have one halal logo because of the number of HCBs (>400 worldwide) as well as the differences in the halal standards used in different countries. Furthermore, to have one halal logo, its creation must be through meetings, negotiations, and agreements by all the main HCBs in the world. Three of the HCBs in this study reported that the lack of one unified halal standard is increasing production costs and complexity of certification, a problem for an HCB and exporting companies, particularly if the halal certifier is following different halal standards. Following multiple halal standards requires accreditation by different accreditation bodies, which costs the HCB more money and the companies that seek halal certification pay more, therefore, the consumer ultimately ends up paying the cost of certification.

Also, HCBs certify products based on the halal standard accepted by the importing countries. For example, Malaysia only accepts halal products certified by an HCB that uses Malaysian halal standard in their certification. Some importing countries through their accreditation bodies have imposed more burdensome requirements, some of which are probably not needed, such as requiring a veterinarian on staff in the HCB. These requirements do not necessarily complicate certification, but they may add undue burdens. All HCBs in this study believed there are some halal standards less stringent than others (e.g., some halal standards accepted forms of animal stunning and some not at all; some accepted mechanical slaughter for poultry; and some just accepted hand slaughter; and so on). Three HCBs believed the reason for choosing or following a particular halal standard is the importing country, which informs which halal standard to follow.

All HCBs agreed it is necessary to have one universal halal standard with a minimum set of standards followed by everyone. The best strategy to do so is to bring the HCB together to discuss the points of difference and come to an agreement with all. Examples of this being done in other U.S. food industry sectors includes the Conference for Food Protection and the Interstate Shellfish Sanitation Conference. The HCBs suggest it is possible to establish one halal standard in the United States, just not one halal logo. However, that task would require a lot of work, time, money, and probably needs to be facilitated by a federal governmental agency in the U.S. Four HCBs believed that the Organization of Islamic Cooperation (OIC) could develop one unified, global halal standard. However, OIC countries have been working on this for years. Achieving this requires greater cooperation by decision-making and influential countries, such as Turkey, Malaysia, Saudi Arabia, and the United Arab Emirates. Nearly all HCBs agreed that establishing a national halal hub in the U.S. is a big challenge because it is more of a business and political issue, not a religious issue. However, one interviewee stated that it is impossible to establish a national halal hub in the U.S. because the national hub must be centrally located among the main consumers (Muslims community) and their number is limited in the United States, about 3.45 million Muslims in the U.S. or 1% of the total U.S. population [38]. Also, he said the United States is geographically removed from the primary halal-consuming regions.

### Limitations

Refusal of 5 of the 11 U.S. HCB to participate in the study was the main limitation in the study. Additionally, using only an online search could have missed some HCBs, which was a study limitation. These HCBs might have different opinions from the participating HCB, impacting the generalizability of the study results to U.S.-based HCB. Interviewing just one person at each HCB for a total of six people suggests that not even each agency’s ideas will be fully represented. Lastly, the authors’ interpretation may also limit the work.

## Conclusions

The main challenge for the halal meat industry is that Muslim consumers have different acceptance of halal standards, as a result of different Islamic denominations. The study findings confirmed that food safety and halal principles are verified by the HCB in the U.S. Applying different halal standards for U.S. HCB makes it challenging for Muslim consumers to determine the standards with which the product was certified. Slaughtering animals is one of the most controversial issues that associated with: slaughterman (Muslim/Christian/Jewish); stunning (acceptable or not); and mechanical slaughter or hand slaughter which is difficult for the Muslim consumer to distinguish between these conditions. The harmonization of the halal standard among the OIC countries is important to ensure the smooth implementation of the halal standard without any misunderstanding or confusion of HCB worldwide. This is certainly in the interest of the Muslim consumer to reduce the cost of certificates and increase consumer confidence. The findings of this study could be beneficial to the halal industry by highlighting the challenges and issues among U.S. HCBs.

## Supporting information

S1 File Interview questions. (DOCX)

S2 File Inclusion criteria for selecting HCB firms. (DOCX)
