# Supplementary material for: Perceived challenges in implementing halal standards by halal certifying bodies in the United States
Source: PLoS One. 2023 Aug 31;18(8):e0290774. doi: 10.1371/journal.pone.0290774 (PMC10470877; doi:10.1371/journal.pone.0290774)
Supplement: S1 File — (DOCX) [file pone.0290774.s001.docx]

**INTERVIEW QUESTIONS**

**Organizational characteristics:**

1. When was your certifying body created?
2. Is your organization accredited?
   1. If yes, by which body or bodies?
   2. Why did you choose this Accreditation Body?
3. Which types of companies apply for halal certification from your firm?
4. Abattoir red meat.
5. Abattoir poultry.
6. Food processing meat.
7. Food processing non-meat (perishable and non-perishable).
8. Chemicals.
9. Cosmetics.
10. Other.
11. Who is eligible or qualified to conduct halal audits in the companies that your firm certifies?
12. How many auditors are required for a single audit?
13. What level of education are they required to have?
14. How long is the duration of halal certification process (from receiving the application to issue halal certificate) to be approved?
15. How long is the halal certificate that you issue valid?
16. How many unique halal certificates did you issue in 2018?

**Role of halal certification bodies in ensuring implementing food safety practices in halal industries.**

1. Do you think halal certifying bodies should be responsible for making sure halal food products are safe? Why?
2. Are food safety violations recorded when your auditors are performing an on-site audit?
   1. Does this effect on the process of issuing halal certificate?
   2. Why?
3. Some segments of the food industry are required to have HACCP Plans. The following questions apply to those businesses you provide halal certification that are required to have a HACCP Plan.
   - 1. Is verification of the HACCP Plan part of the halal certification process? If yes, is this required by halal standard or international standard?
     2. Do you verify the company that seeks halal certificate if it complies to the USDA/FDA regulations in term of food safety?
     3. A HACCP system requires implementation of prerequisite programs. Which prerequisite programs are required by Halal Assurance System?

1. Sanitation Standard Operating Procedures (SSOP).

2. Standard Operating Procedures (SOPs).

3. Good Manufacture Practices (GMP).

1. Laboratory tests are required for verification of microbiological status of food products. Does the Halal Assurance System implementation require performing microbial test?
2. What are these tests?
3. How often are these tests required?
4. Who is collecting samples?

**Issues and challenges of applying different halal standards:**

1. What are the major halal standards that your firm applies? Can you provide us with a list of the standards?
2. Are the forged or fake halal certificates an issue in the United States?
   1. If yes, why do you think this is an issue?
3. Is the use of an expired halal logo a big issue in the United States?
   1. If yes, why is this an issue?

**Challenges to unifying halal standards:**

1. Various halal logos are used in the United States. Do you think the lack of a universal halal standard is the reason?
   1. If yes, why?
   2. If no, what is the reason? (Your opinion).
2. Do you think it is possible to have one logo?
   1. If no, why not?
   2. If yes, why?
3. Do you believe the lack of a unified halal standard is increasing production costs for exporting companies? If yes, why?
4. Is the lack of a unified halal standard increasing complexity for the HCB and exporting companies (problems during issuing halal certificate such as confusion and disputing, and complication after exporting)?
5. Do you believe some halal standards flexible more than other? Explain.
6. What are the main factors that contribute to choosing or following a particular halal standard from other?

**The possibility of cooperation all halal certifying bodies in the United States to unify applying one comprehensive halal standard sponsored by accredited body:**

1. Do you think there should be one halal standard? Why or why not?
2. Do you believe it is possible to establish one halal standard in the United States under the umbrella of accredited body? Why or why not?
3. Do you believe the Organization of Islamic Cooperation (OIC) or International Halal Integrity Alliance (IHI Alliance) can develop unified halal standard globally? Why or why not?
4. What are the challenges to establish a National Halal hub in the United States? (to be the central trading hub for halal products in the United States (50 States) and providing a credible platform in connecting global halal supply chain and certification.
