# Supplementary material for: Perceived challenges in implementing halal standards by halal certifying bodies in the United States
Source: PLoS One. 2023 Aug 31;18(8):e0290774. doi: 10.1371/journal.pone.0290774 (PMC10470877; doi:10.1371/journal.pone.0290774)
Supplement: S2 File — (DOCX) [file pone.0290774.s002.docx]

**Inclusion Criteria**

**Inclusion criteria are those characteristics that qualify prospective HCB firms in our study.**

1. Muslims run the business.
2. Location of the halal certifying bodies (HCBs) within the United States.
3. HCBs are legally permitted (government license).
4. Halal certification was provided by HCBs for a variety of products, including food.
5. Conducting an on-site audit prior to granting a halal certificate.
6. HCB firm’s website availability includes a physical address and full contact information.
